# The many faces of pseudomyxoma peritonei: a radiological review based on 30 cases

**DOI:** 10.1590/0100-3984.2019.0044

**Published:** 2019

**Authors:** Cássia Fonseca, Saulo Carvalho, Teresa Margarida Cunha, Rui Tiago Gil, Nuno Abecasis

**Affiliations:** 1 Instituto Português de Oncologia de Lisboa Francisco Gentil, Lisboa, Portugal.

**Keywords:** Pseudomyxoma peritonei, Tomography, X-ray computed, Neoplasms, cystic, mucinous, and serous/pathology

## Abstract

**Objective:**

To determine the most common imaging features of pseudomyxoma peritonei (PMP), as well as the histologic subtypes of the primary tumors.

**Materials and Methods:**

We reviewed 30 cases of women with pathologically confirmed PMP. Only computed tomography scans were available. All cases were retrospectively studied by four radiologists, working independently. We identified the most common imaging findings, the predominant primary site of the disease, and the growth pattern. The most common sites of recurrence were also analyzed.

**Results:**

The most common computed tomography finding was peritoneal/omental nodules (including “omental caking”), followed by visceral scalloping and non-mucinous ascites. The most common site of the primary tumor was the appendix (in 63.3%), followed by the ovaries (in 16.6%), and 16.6% of the tumors were of undetermined origin. There was one case of synchronous appendiceal and ovarian tumors. Low-grade mucinous neoplasm was the most common histologic subtype, accounting for 84.2% of the appendiceal tumors and 40% of the ovarian tumors.

**Conclusion:**

Although PMP is a relatively rare entity, radiologists must be aware of its possible imaging findings, common locations, and possible patterns of recurrence. The origin of the primary tumor should also be investigated. Future studies are needed in order to determine which preoperative imaging findings predict surgical outcomes and to characterize the main findings of radiological recurrence.

## INTRODUCTION

Pseudomyxoma peritonei (PMP) is a clinical syndrome characterized by the accumulation of mucinous ascites within the peritoneal cavity^(1,2)^. PMP can include mucinous ascites, peritoneal nodules, omental nodules, and ovarian involvement^(3)^. It is caused by primary low-grade mucinous tumors that arise from different sites, usually from the appendix or ovary^(4)^. Recent studies have indicated that virtually all cases originate from the appendix^(5)^. There are many controversies surrounding appendiceal neoplasms and PMP, stemming from the use of inconsistent histologic criteria for their diagnosis and philosophical differences of opinion regarding their pathogenesis^(6,7)^. The coexistence of appendiceal and ovarian mucinous tumors typically manifests as ascites in PMP, the majority of such cases being of appendiceal origin with ovarian implants^(4,8)^.

Correctly diagnosing PMP is essential. The correct staging of PMP is of great importance if an aggressive surgical approach is to be taken^(1,9)^. Surgical reduction (debulking) of the tumor and surgery combined with hyperthermic intraperitoneal chemotherapy, with or without chemotherapy in the immediate postoperative period, is recommended at the majority of centers specializing in the management of PMP^(1,10-12)^.

Imaging is frequently the first step in the diagnosis and management of PMP, with a significant impact on the prognosis^(12,13)^. Therefore, radiologists should be familiar with the imaging features of the disease.

In the present paper, we report the pre- and post-treatment radiological features and pathological analysis of cases of PMP treated at a referral center for cancer.

## MATERIALS AND METHODS

This was a retrospective review of 30 cases of PMP with a definitive pathological diagnosis, all of them in women, identified from the database of a referral center for cancer, treated between 2003 and 2017. Abdominal and pelvic computed tomography (CT) scans were reviewed, as were the results of the pathological analyses. All procedures were performed in accordance with the World Medical Association Declaration of Helsinki, and the study was approved by the local institutional review board.

CT images were retrospectively reviewed by four radiology specialists (with 4-20 years of experience in abdominal imaging), working independently, in order to characterize the signs of PMP; the predominant areas of disease in the peritoneal cavity; the presence and histology of primary tumors; and the signs of recurrent disease after treatment. The presence or absence of the following features was also noted: appendiceal and ovarian tumors; regions of mucinous ascites in the peritoneal cavity and its characteristics (morphology, volume, predominant areas, and septa or calcification in the mucinous material); visceral scalloping; peritoneal or omental nodules; and distant metastases. Disagreements were resolved by consensus.

All CT images had been acquired in one of two 16-slice scanners (Somatom Scope or Somatom Sensation; Siemens Medical Systems, Erlangen, Germany). The contrast medium-iobitridol (Xenetix 350 mg/mL; Guerbet, Aulnay-sous-Bois, France) or iopromide (Ultravist 300 mg/mL; Bayer Schering Pharma, Berlin, Germany)-was administered orally (1000 mL) or intravenously (90-120 mL). Images were acquired in the portal venous phase, with a slice thickness of 5 mm and an interslice gap of 2 mm. Three CT examinations were performed without contrast administration due to a patient history of allergy or patient refusal.

Of the 30 patients evaluated, 15 underwent surgical cytoreduction with hyperthermic intraperitoneal chemotherapy, the remaining patients being treated with surgery only. All of the surgical specimens had been analyzed in the pathology department of the referral center, and the histologic subtypes of the primary tumors had been determined.

## RESULTS

All of the cases in our series were cases of histologically verified PMP. The mean age of the patients at diagnosis was 56.8 years (range, 39-81 years). The imaging findings were variable, ranging from a few peritoneal nodules to abundant mucinous ascites, filling the peritoneal cavity completely or almost completely, also referred to as large volume disease^(14)^.

The morphological aspects of PMP on CT are shown in Table 1, and the main disease locations within the peritoneal cavity are summarized in Table 2. Of the 30 patients evaluated, 19 (63.3%) had primary tumors of appendiceal origin, whereas 5 (16.6%) had primary tumors of ovarian origin (3 in the left ovary and 2 in the right ovary), and the origin was undetermined in 5 (16.6%).

**Table 1:** CT features of PMP.

Feature	(N = 30)
Visceral scalloping, n (%)	13 (43.3)
Ascites, n (%)	12 (40)
Peritoneal or omental nodules, n (%)	17 (56.6)
Omental caking , n (%)	6 (20)
Appendiceal tumor, n (%)	4 (13.3)
Left ovarian mass, n (%)	6 (20)
Right ovarian mass, n (%)	6 (20)
Calcification, n (%)	3 (10)
Septa, n (%)	1 (3.3)

**Table 2:** Distribution of PMP and visceral involvement on CT.

Site	(N = 30)
Right subphrenic space, n (%)	5 (16.6)
Surface of liver, n (%)	10 (33.3)
Left subphrenic space, n (%)	4 (13.3)
Surface of spleen, n (%)	3 (10)
Morrison's pouch, n (%)	1 (3.3)
Right paracolic gutter, n (%)	6 (20)
Left paracolic gutter, n (%)	6 (20)
Pouch of Douglas, n (%)	5 (16.6)
Right colon, n (%)	3 (10)
Right ovary, n (%)	6 (20)
Left ovary, n (%)	6 (20)
Appendiceal tumor, n (%)	4 (13.3)
Stomach, n (%)	3 (10)
Jejunum or ileum, n (%)	1 (3.3)
Small bowel mesentery, n (%)	2 (6.6)
Abdominal wall, n (%)	1 (3.3)

The most common histologic subtype was low-grade mucinous tumor, which was identified in 84.2% of the appendiceal tumors and 40% of the ovarian tumors. Of the five primary ovarian tumors, three were found to be of the borderline mucinous histologic subtype. There was one case of synchronous appendiceal and ovarian tumors that were also of the borderline mucinous histologic subtype. In three of the five cases of undetermined origin, the histologic subtype was low-grade mucinous tumor, whereas the other two (6.6% of the sample as a whole) were of undetermined histologic subtype. Overall, 21 (70.0%) of the 30 tumors were of the low-grade mucinous subtype, 7 (23.3%) were of the borderline mucinous subtype, and 2 (6.6%) were of an undetermined histologic subtype. On follow-up CT scans acquired at 3-12 months after treatment (Table 3), we detected signs of recurrent disease in 19 patients (63.3%).

**Table 3:** Features of PMP on follow-up CT.

Feature	(N = 19)
Visceral scalloping, n (%)	9 (47.3)
Ascites, n (%)	7 (36.8)
Peritoneal or omental nodules, n (%)	16 (84.2)
Adrenal nodule, n (%)	4 (21)
Thoracic lesion, n (%)	3 (15.7)
Calcification, n (%)	1 (5.2)

**Figure 1:**
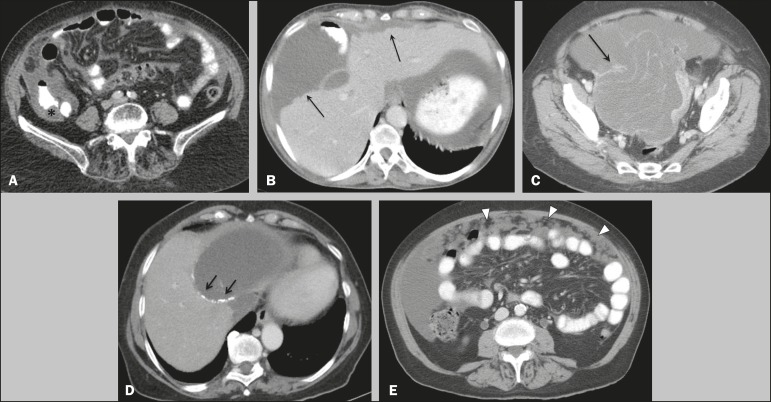
Morphological patterns of PMP. Appendiceal primary tumor (asterisk) accompanied by pelvic mucinous ascites (**A**); liver scalloping (arrows in **B**); septa (arrow) in pelvic mucinous ascites (**C**); peripheral calcifications (arrows in **D**); multiple omental nodules with an omental caking aspect (arrowheads) and ascites in the right paracolic gutter (**E**).

**Figure 2:**
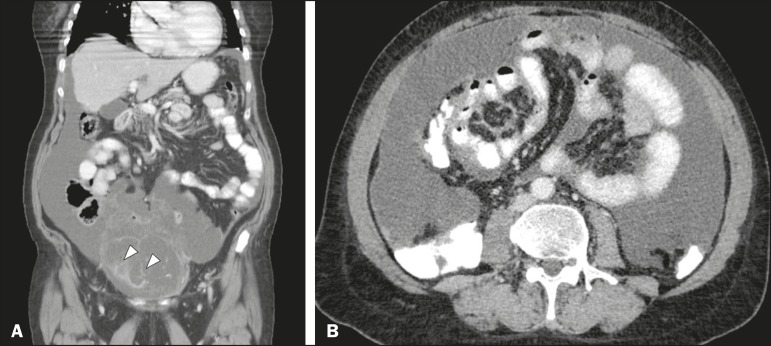
Mucinous ascites on the pelvis, paracolic gutters and bilateral subdiaphragmatic recesses, with septations in the pelvis (arrowheads in **A**); large volume disease with central displacement of the small bowel (**B**).

**Figure 3:**
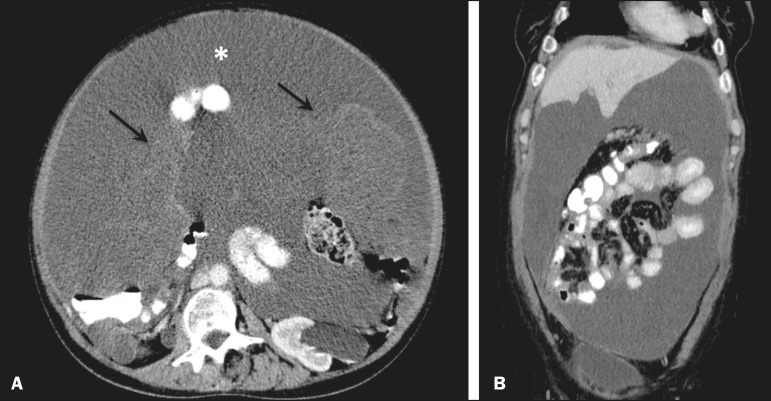
Mucinous ascites with high-attenuation elements forming bulky deposits (arrows), in contrast with low-attenuation ascites (asterisk) in large volume disease (**A**); large volume disease characterized by lowattenuation ascites sparing the mesentery and displacing the intestinal loops centrally (**B**).

**Figure 4:**
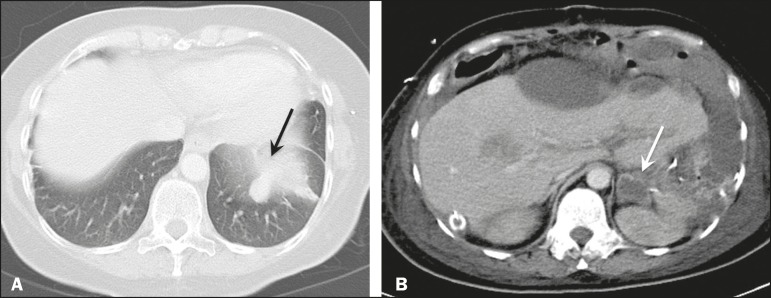
Follow-up CT scans of two different patients showing metastases to the lung (arrow in **A**) and to the left adrenal gland (arrow in **B**).

**Figure 5:**
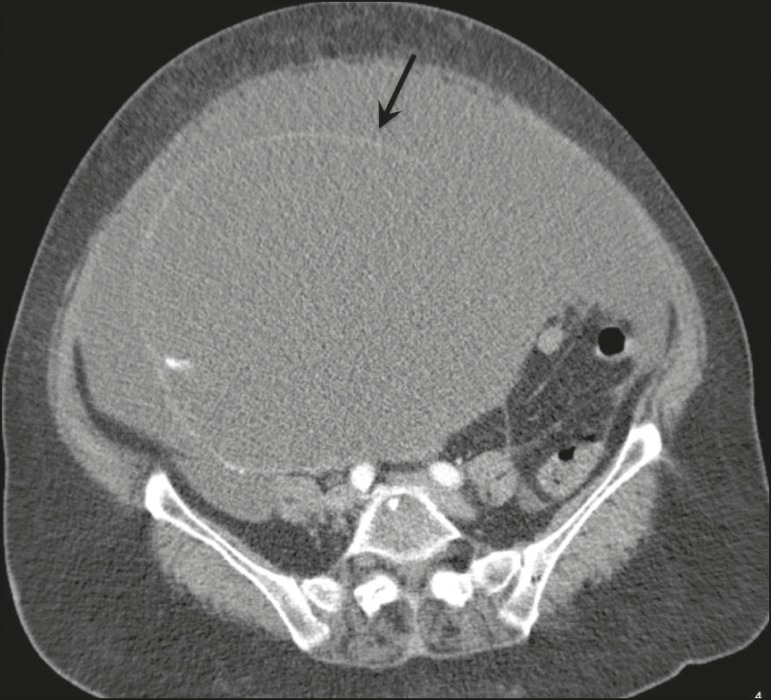
Primary ovarian mucinous borderline tumor (arrow) in a patient with large volume disease.

In most cases, the primary tumor was not detected by the radiologists in the initial CT study, who instead reported mainly peritoneal/omental nodules (including “omental caking”), visceral scalloping, and non-mucinous ascites (Figure 1). The volume of disease was greater on the surface of the liver and in the right subphrenic space, reflecting the pathways of flow of intraperitoneal fluid (Figure 2). The tumors tended to spare mobile loops of the small bowel, instead accumulating at other sites, such as the pelvis, paracolic gutters, omentum, and liver capsule (Figure 3). As depicted in Figure 4, there were also cases in which follow-up CT scans showed signs of adrenal metastases (in 21%) and pulmonary metastases (in 15.7%). In cases of large volume disease (Figure 5), it was more difficult to recognize the primary tumor.

## DISCUSSION

CT imaging plays an important role in the detection of mucocele of the appendix, which is a significant prognostic factor, allowing surgeons to take the necessary precautions to avoid perioperative rupture and the consequent peritoneal seeding^(13)^. Even in cases of early detection of rupture of a mucocele, a more tailored and radical approach could be taken, reducing the chances of reoperation with unfavorable prognoses.

Because of the relatively low incidence of PMP, there have been few studies detailing its CT characteristics. Sulkin et al.^(14)^ described CT features in 17 cases, and Walenski et al.^(15)^ presented a pictorial essay based on 21 cases, both studies including cases in men and women. In addition, there have been a few case reports^(4,16,17)^, which also included patients of both genders. In the present study, we had access to pre- and post-treatment CT examinations of 30 patients with PMP, all female, in order to assess pathologically proven ovarian and appendiceal tumors.

The classic signs of PMP on CT include low-attenuation lesions or loculated ascites scattered throughout the peritoneal cavity and individual peritoneal or omental nodules. These implants often have a characteristic mass effect on the liver and spleen, producing a scalloped appearance, which is the most representative sign of PMP on CT^(18-20)^. When there is a large volume of disease, thick, voluminous low-attenuation ascites, with or without septa and calcification, can be present^(14,16,21)^, impeding the evaluation of the primary tumor, which is often masked by the copious amount of fluid in the peritoneal cavity. Our study corroborates those findings, showing that in most cases the primary tumor was not detected by the radiologists in the initial CT study.

The pattern of mucinous ascites distribution seen in the present study is in agreement to what has been described previously^(22-24)^, the volume of disease being greater on the surface of the liver and in the right subphrenic space, reflecting the pathways of flow of intraperitoneal fluid. The mucus and the cells it contains follow the normal flow of peritoneal fluid and are “redistributed” within the peritoneal cavity to sites of fluid absorption through lymphatic lacunae and lymphoid aggregates. Consequently, the tumor tends to spare mobile loops of the small bowel. Bulky accumulations can form as the mucus is absorbed, during which epithelial cells are “filtered out” and concentrated^(7,8)^. In our patient sample, the main sign of recurrence seen on CT was peritoneal or omental nodules, followed by visceral scalloping and low-attenuation ascites. As previously mentioned, there were also cases in which follow-up CT scans showed signs of adrenal metastases and pulmonary metastases, both of which are rare findings^(25)^.

The tumor grade is considered one of the most important prognostic factors in PMP^(11,26)^. Typically, the selection of patients to undergo more aggressive surgical therapy takes this into account. It has also been shown that complete cytoreduction is associated with increased survival. In a study conducted at a large cancer center, Miner et al.^(11)^ stated that it is very difficult to determine whether better survival rates are due to treatment, tumor biology, or patient screening, and that categorical statements regarding the topic are therefore discouraged.

Similar to Smeenk et al.^(27,28)^, we found that the low-grade mucinous pattern was the most common histological subtype among primary appendiceal tumors. In addition, seven of our cases were described as likely primary ovarian tumors in the imaging examination but were confirmed as metastatic involvement in the pathological analysis, which is in agreement with the notion that most cases of ovarian tumors in PMP represent metastases from appendiceal mucinous tumors^(3,23,26,29)^.

The main limitations of our study are its retrospective nature and the fact that the images were obtained at different stages of the disease. PMP is a complex entity, with various forms of presentation, and its management therefore varies until the definitive diagnosis is made. In addition, we had no access to the patients or their clinical histories. Furthermore, it was difficult to recognize the primary tumor in cases of large volume disease. Moreover, not all patients underwent surgical treatment at the same institution, which impeded the adequate evaluation of recurrence.

In conclusion, we have reviewed the CT findings of 30 women with PMP seen at a referral center for cancer, listing the main imaging features and describing the predictable pattern of peritoneal flow distribution. In addition to the visceral scalloping that is specific to PMP, radiologists must also be aware of its other CT features, such as ascites and omental nodules. Furthermore, possible primary tumors should be investigated, especially in small volume disease. In large volume disease, CT findings can overlap and are more nonspecific. There is a need for additional studies in order to improve the ability to predict surgical outcomes on the basis of preoperative imaging and to characterize the main findings of radiological recurrence.
